# Probiotic Mixture Attenuates Colorectal Tumorigenesis in Murine AOM/DSS Model by Suppressing STAT3, Inducing Apoptotic p53 and Modulating Gut Microbiota

**DOI:** 10.1007/s12602-024-10405-1

**Published:** 2024-12-06

**Authors:** Hoi Kit Matthew Leung, Emily Kwun Kwan Lo, Congjia Chen, Fangfei Zhang, Felicianna, Marsena Jasiel Ismaiah, Hani El-Nezami

**Affiliations:** 1 School of Biological Sciences, University of Hong Kong, Pokfulam, Hong Kong, 999077 China; 2 Institute of Public Health and Clinical Nutrition, School of Medicine, University of Eastern Finland, 70211 Kuopio, Finland

**Keywords:** Prohep, Probiotic mixture, Colorectal cancer, Gut microbiota, Acetate

## Abstract

Colorectal cancer (CRC) is one of the most common cancers worldwide. The standard CRC chemo drug, 5-Fluorouracil (5-FU), has a poor response rate and chemoresistance, prompting the need for a more effective and affordable treatment. In this study, we aimed to evaluate whether Prohep, a novel probiotic mixture, would alleviate azoxymethane/dextran sodium sulfate (AOM/DSS)-induced colorectal tumorigenesis and enhance 5-FU efficacy and its mechanism. Our results suggested that Prohep showed stronger anti-tumorigenesis effects than 5-FU alone or when combined in the AOM/DSS model. Prohep significantly reduced the total tumor count, total tumor size, caecum weight, colonic crypt depth, colonic inflammation, and collagen fibrosis. Prohep downregulated pro-inflammatory TNF-α and proliferative p-STAT3 and upregulated apoptotic p53. Metagenomics analysis indicated that Prohep-enriched *Helicobacter ganmani*, *Desulfovibrio porci*, *Helicobacter hepaticus*, and *Candidatus Borkfalkia ceftriaxoniphila* were inversely correlated to the total tumor count. In addition, Prohep-enriched *Prevotella* sp. *PTAC* and *Desulfovibrio porci* were negatively correlated to AOM/DSS enriched bacteria, while forming a co-existing community with other beneficial bacteria. From KEGG analysis, Prohep downregulated CRC-related pathways and enhanced pathways related to metabolites suppressing CRC like menaquinone, tetrapyrrole, aminolevulinic acid, and tetrahydrofolate. From Metacyc analysis, Prohep downregulated CRC-related peptidoglycan, LPS, and uric acid biosynthesis, and conversion. Prohep elevated the biosynthesis of the beneficial L-lysine, lipoic acid, pyrimidine, and palmitate. Prohep also elevated metabolic pathways related to energy utilization of lactic acid-producing bacteria (LAB) and acetate producers. Similarly, fecal acetate concentration was upregulated by Prohep. To sum up, Prohep demonstrated exceptional anti-tumorigenesis effects in the AOM/DSS model, which revealed its potential to develop into a novel CRC therapeutic in the future.

**Graphical Abstract:**

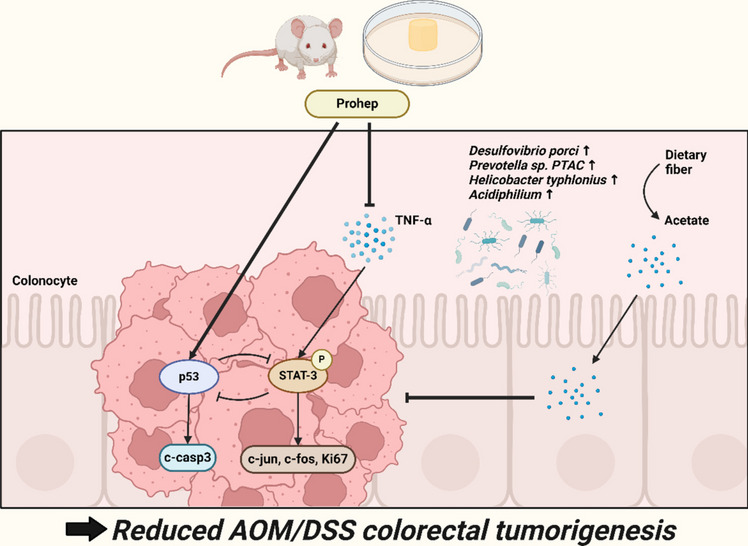

**Supplementary Information:**

The online version contains supplementary material available at 10.1007/s12602-024-10405-1.

## Introduction

Colorectal cancer (CRC) is the third most diagnosed cancer, as well as the second leading cause of cancer death worldwide [1, 2]. Over half of the cases are accounted for risk factors including smoking, an unhealthy diet, high alcohol consumption, physical inactivity, and excess body weight [3]. The diagnosis of CRC is often at later stages, and its 5-year survival rate at the advanced stage is only 15%, indicating the importance of early diagnosis and effective treatment [4]. Surgical treatments, including endoscopic mucosal resection and endoscopic submucosal dissection in the early stages and lymph node dissection in the advanced stages along with polyps removal, are the standard first-line treatment for CRC [5]. Apart from surgical procedures, chemotherapeutic intervention is crucial to prevent and cure metastatic CRC and improve patients’ survival rates [6]. Nonetheless, the response rate of the standard chemo drug backbone 5-fluorouracil (5-FU) on CRC is as low as 11% without combining with other regimens [7]. Given the high economic burden of chemotherapy [8], affordable and effective pharmaceutical options are in demand to save lives.

An emerging direction for researching novel CRC treatments is microbiome-targeted therapy [9–11]. Gut microbiota and cancer exhibit a bidirectional relationship, in which the alteration of microbial composition could promote pathogenesis, while the modulation of gut microbiota could also be therapeutic for cancer [12, 13]. Gut microbiota’s influence on CRC initiation and progression was suggested to be both the “driver” and the “passenger” of the “driver/passenger” theory [14]. The imbalance of the gut microbiota profile termed gut dysbiosis was found to be a vital cause of colorectal tumorigenesis [14, 15]. On the contrary, modulation of the gut microbiota alleviated or prevented the progression of CRC in both healthy and cancerous subjects [12, 13]. Microbiome-targeted therapy in CRC treatment includes fecal microbiota transplant (FMT), probiotics, diet and prebiotics, and antibiotic treatment [9–11]. Unlike the complexity and high cost of personalized FMT, the damaging effects of antibiotics to commensal bacteria, the induction of probiotics in CRC treatment is a more affordable and efficient option for modulating the gut microbial composition [9–11]. Evidence from studies has shown that probiotics are a successful treatment for colorectal cancer (CRC) by limiting the growth of pathogens, reversing dysbiosis, modulating the immune system, strengthening the intestinal barrier, and promoting an anti-cancer cell signaling network [16, 17]. 5-FU is an essential component in the standard chemotherapy of CRC and yet its efficacy was limited due to the development of chemoresistance and toxicity [18]. Adjuvant combinations of probiotics with 5-FU were found to improve 5-FU applicability in CRC management. When treated with *Lactobacillus* and *Bifidobacterium*, the anti-cancer effects of 5-FU were enhanced with reduced intestinal toxicity and chemoresistance effects [19–21].

Prohep, a novel probiotic mixture, ameliorated high-fat diet (HFD)-induced metabolic dysfunction-associated steatotic liver disease (MASLD), HFD induced metabolic dysfunction-associated steatohepatitis (MASH), and hepatocellular carcinoma (HCC) growth in mice from our previous studies [22, 23]. However, its effect on CRC was not known. We would like to explore if Prohep exerts anti-tumor, anti-inflammatory and immunomodulating effects to alleviated CRC tumorigenesis and whether Prohep would improve 5-FU effectiveness on CRC when working alongside it. This study aimed to investigate the effects of Prohep with and without 5-FU on AOM/DSS-induced colorectal tumorigenesis. The study also aimed to explore the mechanisms by which Prohep would exert its potential anti-CRC effects.

## Methods

### Chemicals and Antibodies

Azoxymethane (AOM) and dextran sulfate sodium (DSS) were purchased from Sigma-Aldrich (St. Louis, MO, USA) and TdB Labs (Uppsala, Sweden), respectively. Prohep probiotic mixture formula composed of *Lactobacillus helveticus* strain *Lh12, Lactobacillus acidophilus (Moro) Hansen* and *Mocquot, Lactobacillus rhamnosus (Hansen), Lactobacillus paracasei* subsp. *Paracasei, Lactobacillus plantarum* subsp. *plantarum (Orla-Jensen)*, *Bifidobacterium animalis* subsp. *Lactis*, *Bifidobacterium breve Reuter*, and *Streptococcus thermophilus Orla-Jensen*. Prohep was produced in lyophilized powder under GMP (Fukopharma, Finland). All primary antibodies were bought from Abcam (Cambridge, UK) and CST (Massachusetts, USA), while the secondary antibodies were purchased from Bio-Rad (Hercules, CA, USA).

### Animals and Eexperimental Setup

The experimental animals were obtained from the Centre for Comparative Medicine Research (CCMR, HKU). 6 weeks old male BALB/c mice were placed in 12-h light/dark cycle with a normal chow diet and drinking water given ad libitum for acclimatization upon the first week of receival. Voluntary gel administration training was carried out for the week afterwards. The chow diet being was taken away overnight on the first day and 0.20 g of MediGel® Sucralose gel (Maine, USA) was given the next morning in individual cages. Mice were allowed to return to their house cages after consuming the given gel. The training continued for another two days but chow diet intake was not constrained. Mice group allocation was as follows: 1. Healthy control (H), 2. AOM/DSS control (AOMDSS), 3. 5-FU (F), 4. Prohep (P) and 5. Prohep + 5-FU (PF). As previously described, the AOM/DSS model was established by injecting the mice with AOM (10 mg/kg) intraperitoneally at the start of the experiment, while PBS was injected for the healthy control group [24]. One week of DSS (2.5%), added in drinking water, was provided and be replaced by regular drink water in the next week. Three cycles of DSS treatment were given in total. Healthy control group received regular drinking water for all time. Prohep administration started at week 0. Prohep (7 × 10^9^ CFU per mice) were infused into MediGel® Sucralose gel (Maine, USA) and provided to each mouse of Prohep and Prohep + 5-FU groups at 0.20 g every other day until the end of the experiment. The dosage of Prohep was based on the previous studies of our group. The other groups received MediGel® Sucralose gel without modification as control. Starting at week 5, 5-FU (35 mg/kg) was injected intraperitoneally to 5-FU and Prohep + 5-FU groups weekly [25]. PBS (vehicle) was injected into other groups. At week 12, the animals were sacrificed, and the colon was cut open longitudinally to determine the tumor count and colon length.

**Fig. 1:**
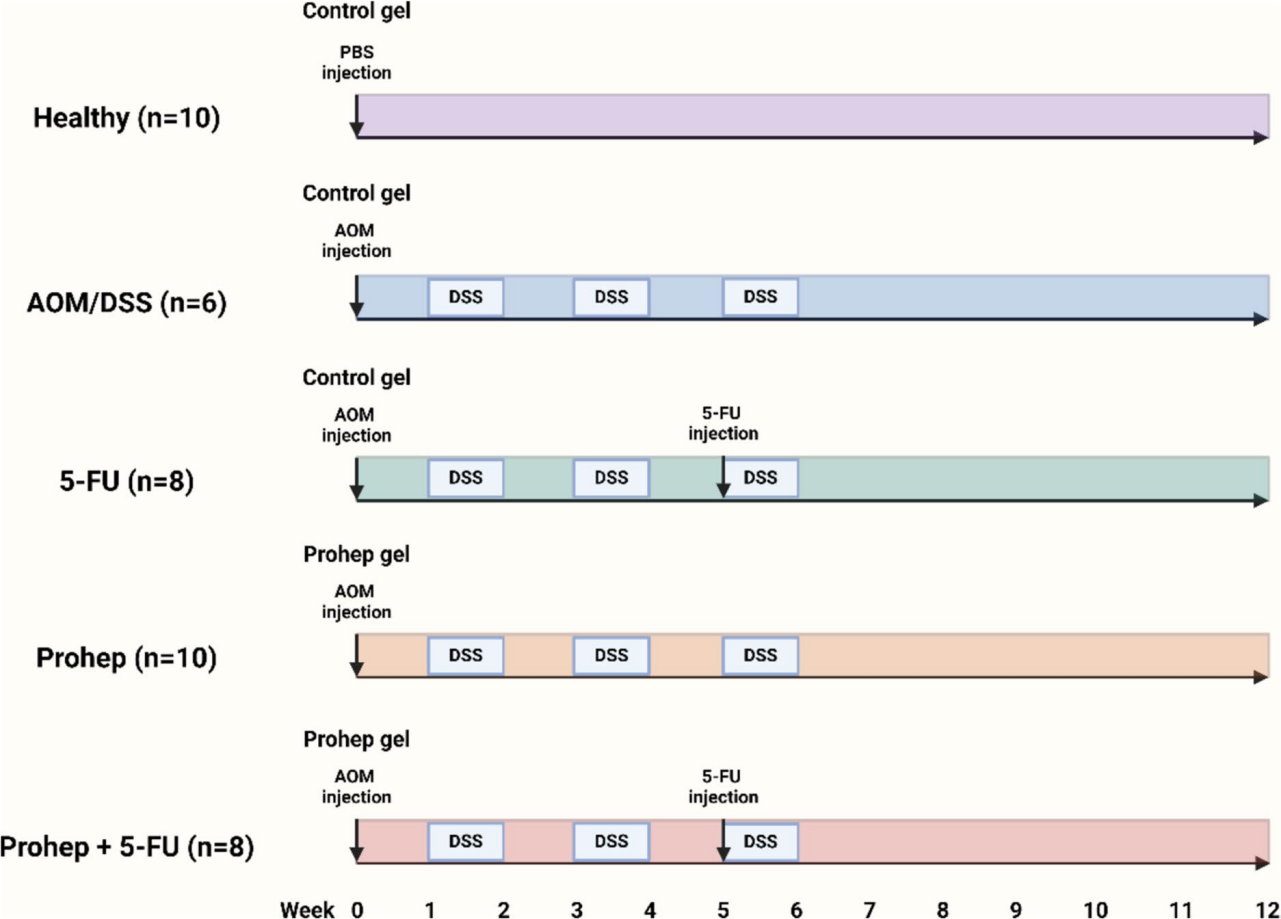
Experimental design of the study. AOM, azoxymethane; DSS, dextran sulfate sodium; and 5-FU, 5-fluorouracil

Colon samples were frozen at − 80 °C for biochemical analysis and were fixed in 10% formalin for histology analysis. Fecal samples were collected and frozen at − 80 °C on the sacrifice day. Liver samples were weighted. All protocols and procedures were approved by the Committee on the Use of Live Animals in Teaching and Research of the University of Hong Kong (CULATR No. 5584–20). Fig. 1

### Colon Histological Analysis

Formalin-fixed colon samples were embedded into paraffin blocks. Specimens were sectioned on slides and deparaffinized to undergo staining procedures. Haematoxylin and eosin (H&E) staining was performed according to the manufacturer’s manual (BASO, Wuhan, China). Adenoma counts were performed with light microscopy. Sirius red staining was carried out according to the manufacturer’s manual (Abcam, Cambridge, UK). The percentage of positive red staining area over the total colon area measured was used to quantify the extent of colonic collagen fibrosis.

### IHC Staining

Immunohistochemistry (IHC) staining was performed with heat-induced antigen retrieval method with sodium citrate (pH6) or Tris–EDTA (pH9). Endogenous peroxidase activity was inhibited with 3% H_2_O_2_, and sections were blocked with CAS-block reagent (Invitrogen, Waltham, MA, USA) for 1 h. Endogenous peroxidase activity was inhibited with 3% H_2_O_2_ and blocked with CAS-block reagent (Invitrogen, Waltham, MA, USA) for 1 h. Primary antibodies incubation (1:100) was performed at 4 °C overnight followed by secondary antibodies incubation (1:250) at room temperature for 1 h. DAB (Abcam, Cambridge, UK) chromogen reaction and hematoxylin counterstaining were performed to visualize the positively stained area. The percentage of positively stained area was determined with ImageJ software (NIH, USA), and histoscore was calculated by multiplying the percentage with the intensity of staining graded from 0, non-stained; 1, weakly stained; 2, moderately stained; and 3, strongly stained [26].

### Cytokines ELISA Analysis

Protein extraction was performed by homogenizing colonic samples in RIPA buffer with protease and phosphatase inhibitor (Sigma-Aldrich, St. Louis, MO, USA), and protein samples were collected by centrifuging the homogenate. The total protein content was measured using DC protein assay (Bio-Rad, CA, USA). ELISA analysis of TNF-α was performed using Mouse ELISA MAX™ Set (BioLegend, CA, USA), according to the instructions of manufacturer. The absorbance was measured using SpectraMax iD3 microplate readers (Molecular devices, CA, USA).

### Western Blot Analysis

Diluted protein was electrophorized in 10% SDS-PAGE gel and transferred to a polyvinylidene fluoride (PVDF) membrane. The membrane was blocked with 5% non-fat milk or BSA, followed by overnight exposure of anti-p53 (1:1000) (Abcam, Cambridge, UK) at 4 °C. Secondary antibody exposure was performed the next day with goat anti-rabbit IgG (H + L) HRP conjugate or goat anti-mouse IgG (H + L) HRP conjugate (1:4000, Bio-Rad, CA, USA) for 1 h at room temperature. Protein bands were visualized with enhanced chemiluminescence reagents (Bio-Rad, CA, USA) using ChemiDox XRS + imaging system (Bio-Rad, CA, USA).

### Metagenomics

Fecal samples obtained on the sacrifice date were used to extract microbial DNA using QIAamp® PowerFecal® Pro DNA kit (Qiagen, Hilden, Germany), according to the manufacturer’s instructions. Extracted DNA was sent to BGI Genomics (Shenzhen, China) for whole genome shotgun sequencing. SOAPnuke was used to filter out adaptor reads that were of low quality or artificial [27]. Bowtie2 [28] was used to exclude the reads mapped to phix and mice contamination (GRCm39) genomes. Using the most recent release (2023–05–10) of the NCBI nr database, which contains archaea, bacteria, viruses, fungi, and microbial eukaryotes, taxonomic profiles were produced using Kaiju [29] with parameter “-e 5.” Taxa excluded from the downstream analysis were those that were either present in less than 10% of the samples or had a relative abundance of less than 0.01%. R package phyloseq [30] was used to aggregate the raw abundance table into a species-level counts per million (CPM) table. R package vegan [31] was then used to determine alpha and beta diversity. Dunn test and PERMANOVA (using adonis2 from the R package vegan) were applied for alpha diversity and beta diversity, respectively, to assess the overall composition difference between groups. ANCOM-BC [32] was used to compare the abundance of microorganisms between AOM/DSS with and without treatment. Linear discriminant analysis (LDA) [33] was also used to lessen the bias present in the various differential methods [34]. Microorganisms that met certain criteria were deemed differentially abundant, including fold change ≥ 2 or ≤ 1/2, LDA score (1og10 transformed) ≥ 2, and *FDR* ≤ 0.05. Concatenated paired-end read files were input to HUMAnN3 (v3.7) in order to evaluate the abundance of metabolic Metacyc pathways and gene families’ abundance [35]. Kyoto Encyclopedia of Genes and Genomes (KEGG) ontology (KO), KEGG module and Metacyc pathway profiles were obtained by utilizing the HUMAnN3 regroup table function. We used ANCOM-BC to assess the differential abundances (DA) of KO and the abundance of metabolic pathways. KO and pathways that met *FDR* value < 0.1 were deemed differentially abundant. Dunn’s post hoc test was employed after the Kruskal–Wallis test for general statistical comparisons between several groups. Spearman’s rank correlation, found in the function cor.test of the R package stat, was utilized for general correlation analysis. The *p*-value was adjusted for multiple testing adjustments using a false discovery rate (*FDR*) (Benjamini–Hochberg). All statistical analyses and visualizations were carried out in the R environment, unless otherwise noted [36].

### SCFA Analysis on Fecal Content

Fecal short-chain fatty acid (SCFA) content was determined using gas chromatography-mass spectrometry (GC–MS) according to previous studies [37, 38]. In short, fecal samples were homogenized in 0.005 M sodium hydroxide with internal standard (10 µg/mL acetic acid-d4) and centrifuged at 13,200 × g for 20 min. The supernatant was then mixed with 0.5 mL of 1-propanol/pyridine (3:2, v/v) and 0.1 mL of propyl chloroformate. The SCFAs were derivatized by vertexing the mixture for 1 min and incubating it at 60 °C. Hexane (0.5 mL) was then added, vortexed, and centrifuged at 2000 × g for 5 min. Of the top layer 400 µL was used for GC–MS analysis (Agilent 6890 N-5973 GC–MS, USA) with conditions based on previous studies [37]. By constructing the calibration curves using the response ratios of acetic acid, propionic acid, and butyric acids against acetic acid-d4, the concentration of the SCFAs was obtained.

### Statistical Analysis

Statistical analysis was performed with GraphPad Prism 8.0 (GraphPad Software, San Diego, CA, USA). All data were presented as *mean* ± standard deviation (*SD*). Student’s *t*-tests or Mann–Whitney *U* tests were adopted to compare differences between two groups. One-way analysis of variance (ANOVA) followed by Tukey’s multiple comparisons test or the Kruskal–Wallis test followed by Dunn’s multiple comparisons test was computed to compare differences in more than two groups. The Spearman correlation coefficient was employed to compute the correlation between two variables. For multiple-testing corrections, a false discovery rate (*FDR*) (Benjamini–Hochberg) was used to adjust the *p*-value. A *p*-value < 0.05 was recognized as having statistical significance.

## Results

### Prohep Significantly Improved Survival and Reduced Colorectal Tumorigenesis of AOM/DSS Mice

**Fig. 2:**
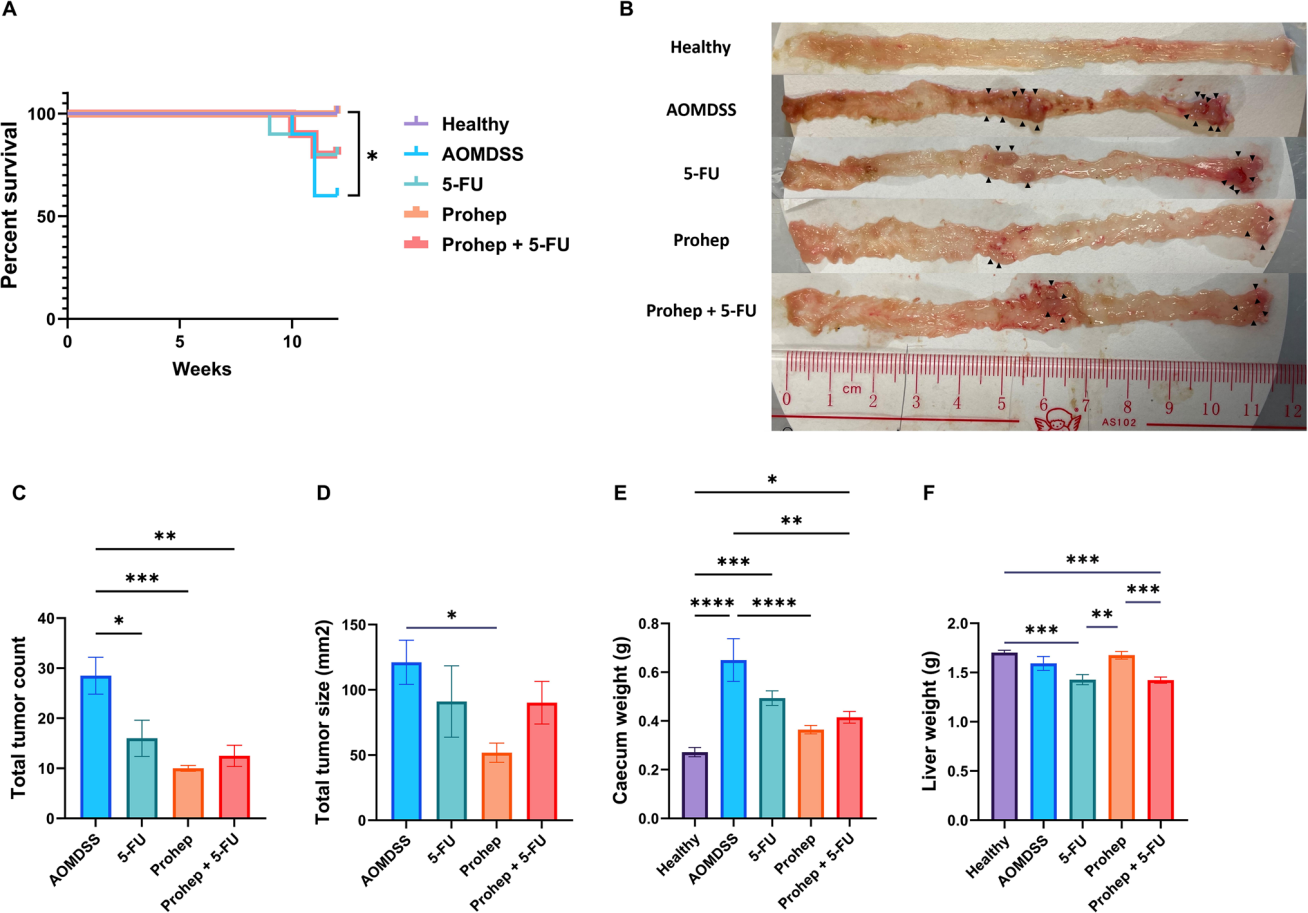
Prohep enhanced survivability and reduced colorectal tumorigenesis of AOM/DSS mice better than 5-FU and Prohep + 5-FU. **A** Survival rate percentage. **B** Representative macroscopic pictures of the dissected colon. **C** Total tumor counts. **D** Total tumor size. **E** Caecum weight. **F** Liver weight. *n* = 6–10. * *p* < 0.05; ** *p* < 0.01; *** *p* < 0.001; **** *p* < 0.0001

Among all treatment groups, only Prohep significantly increased the survival rate against the AOM/DSS group (*p* < 0.05) (Fig. 2A). While 5-FU, Prohep, and Prohep + 5-FU all significantly reduced the total tumor count induced by AOM/DSS (*p* < 0.05), only Prohep group also significantly reduced the total tumor size (*p* < 0.05) (Fig. 2B-D). The caecum weight was elevated in AOM/DSS group (*p* < 0.05). No significant difference in caecum weight between the Prohep group and the healthy control group (*p* > 0.05) was found, indicating that Prohep was effective in preventing caecum enlargement (Fig. 2E). Similar to the 5-FU group, Prohep + 5-FU did not improve survivability and reduce total tumor size, as seen from the Prohep group. In addition, the administration of 5-FU led to the reduction of liver weight in 5-FU and Prohep + 5-FU groups when compared with healthy and Prohep groups (*p* < 0.05) (Fig. 2F).

**Fig. 3:**
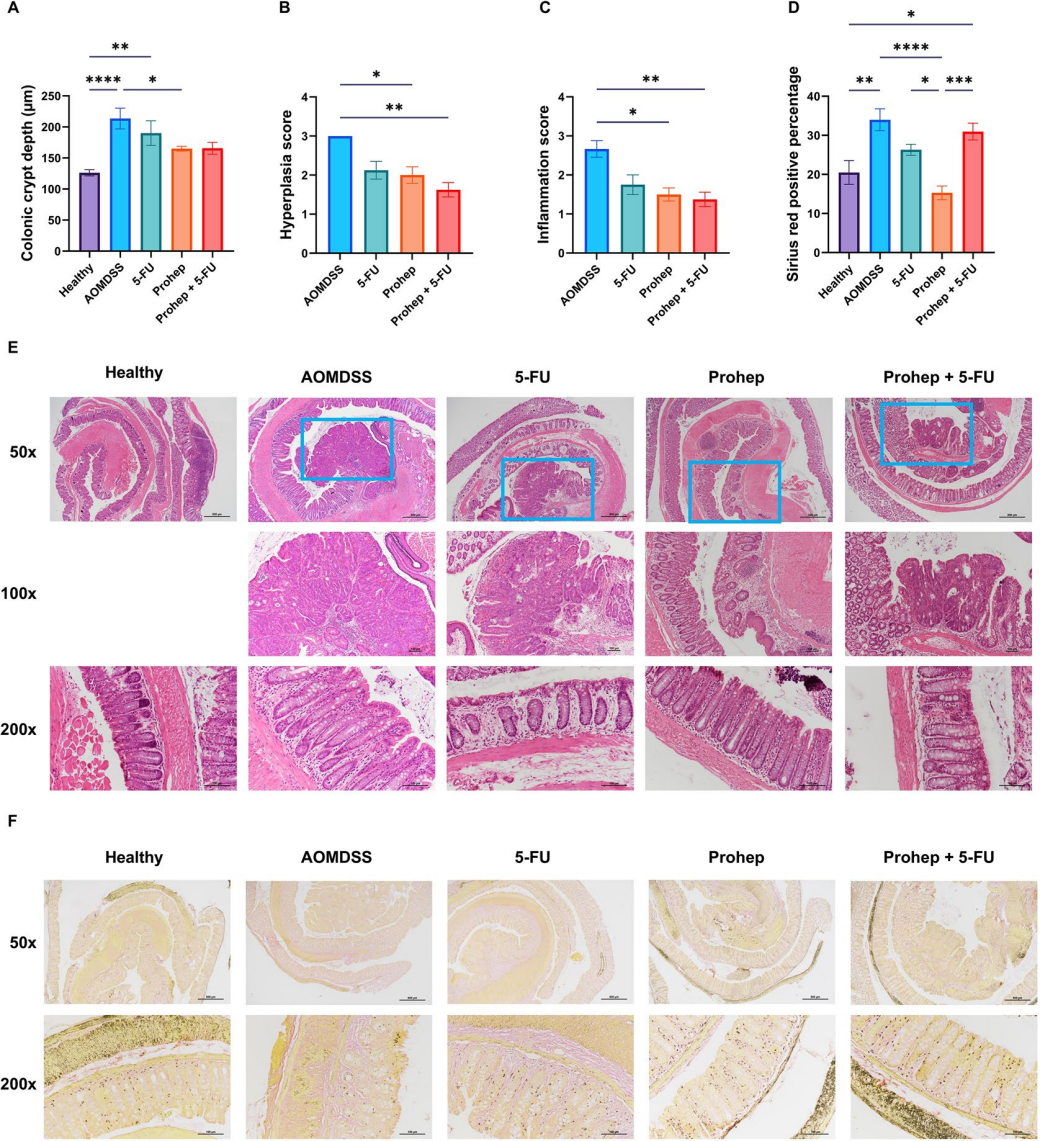
Prohep improved colorectal histopathology induced by AOM/DSS. **A** Colonic crypt depth. **B** Hyperplasia score. **C** Inflammation score. **D** Sirius red positive percentage. **E** Representative microscopic pictures of colonic sections under H&E staining. Colonic tumors were framed in blue rectangle and displayed in 100 × . **F** Representative microscopic pictures of colonic sections under Sirius red staining. *n* = 6–10. * *p* < 0.05; ** *p* < 0.01; *** *p* < 0.001; **** *p* < 0.0001

**Fig. 4:**
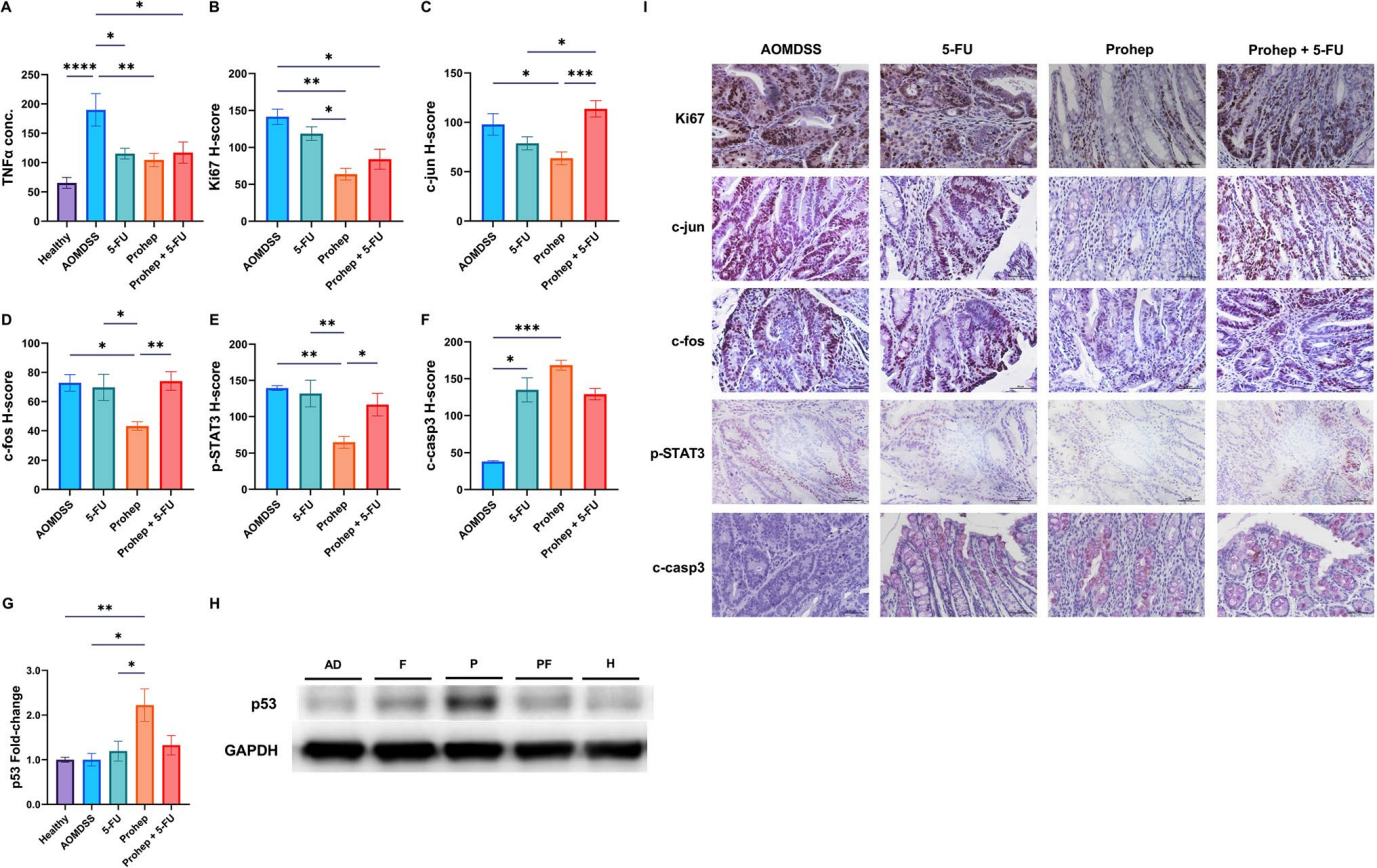
Prohep suppressed pro-inflammatory and proliferative marker and promoted apoptotic markers. Colonic concentration of **A** TNF-α. Immunohistochemistry analysis H-score of **B** Ki67; **C** c-jun; **D** c-fos; *E* p-STAT3; and **F** c-casp3. Western blotting analysis of **G** p53. (H) Representative immunoblots of p53. **I** Representative microscopic pictures of colonic sections under IHC staining in 400 × magnification. *n* = 6–10. * *p* < 0.05; ** *p* < 0.01; *** *p* < 0.001; **** *p* < 0.0001

In terms of histopathology, the elevated colonic crypt depth was reduced by Prohep (p < 0.05), but not 5-FU and Prohep + 5-FU (Fig. 3A). Meanwhile, both hyperplasia and inflammation scores were significantly reduced by Prohep and Prohep + 5-FU correspondingly (*p* < 0.05) (Fig. 3B-C). The reduction effects were not observed in the 5-FU group. From Sirius red staining, Prohep is the only group which alleviated the heightened Sirius red positive percentage (*p* < 0.05), which 5-FU and Prohep + 5-FU could not (Fig. 3D). It indicated that Prohep as the only treatment group to reduce the degree of collagen fibrosis in colon. The proliferative and apoptotic status was evaluated with IHC staining. Prohep and Prohep + 5-FU significantly reduced the H-score of the proliferative marker, Ki67 (*p* < 0.05), but 5-FU did not (Fig. 4B). On the other hand, 5-FU and Prohep improved the H-score of the apoptotic marker, c-casp3 (*p* < 0.05), but not by Prohep + 5-FU (Fig. 4F).

### Prohep Inhibited Colorectal Tumorigenesis Through Suppressing Proliferative p-STAT3 and c-jun/c-fos and Promoting Apoptotic p53

AOM/DSS elevated the levels of pro-inflammatory cytokine, TNF-α (*p* < 0.05) (Fig. 4A). All treatments including 5-FU, Prohep, and Prohep + 5-FU significantly reduced the concentration of TNF-α (*p* < 0.05) (Fig. 4A). Prohep significantly suppressed the level of proliferative p-STAT3, c-jun, and c-fos (*p* < 0.05) (Fig. 4C-E). No suppressive effects on cancer cell progression were observed in the 5-FU and Prohep + 5-FU groups. Although 5-FU also demonstrated elevation in the apoptotic marker, c-casp3 (*p* < 0.05) (Fig. 4F), only Prohep could significantly promoted the protein level of apoptosis inducing p53 (*p* < 0.05) (Fig. 4G, H). No alteration of apoptotic markers was observed from the Prohep + 5-FU group. It was thus suggested that Prohep suppressed STAT3, c-jun, and c-fos and activate p53 to inhibit colorectal tumorigenesis.

### Prohep Modified Gut Microbiota Profile and Promote Biomarkers to Elevate Beneficial Metabolites Biosynthesis and Acetate Concentration

**Fig. 5:**
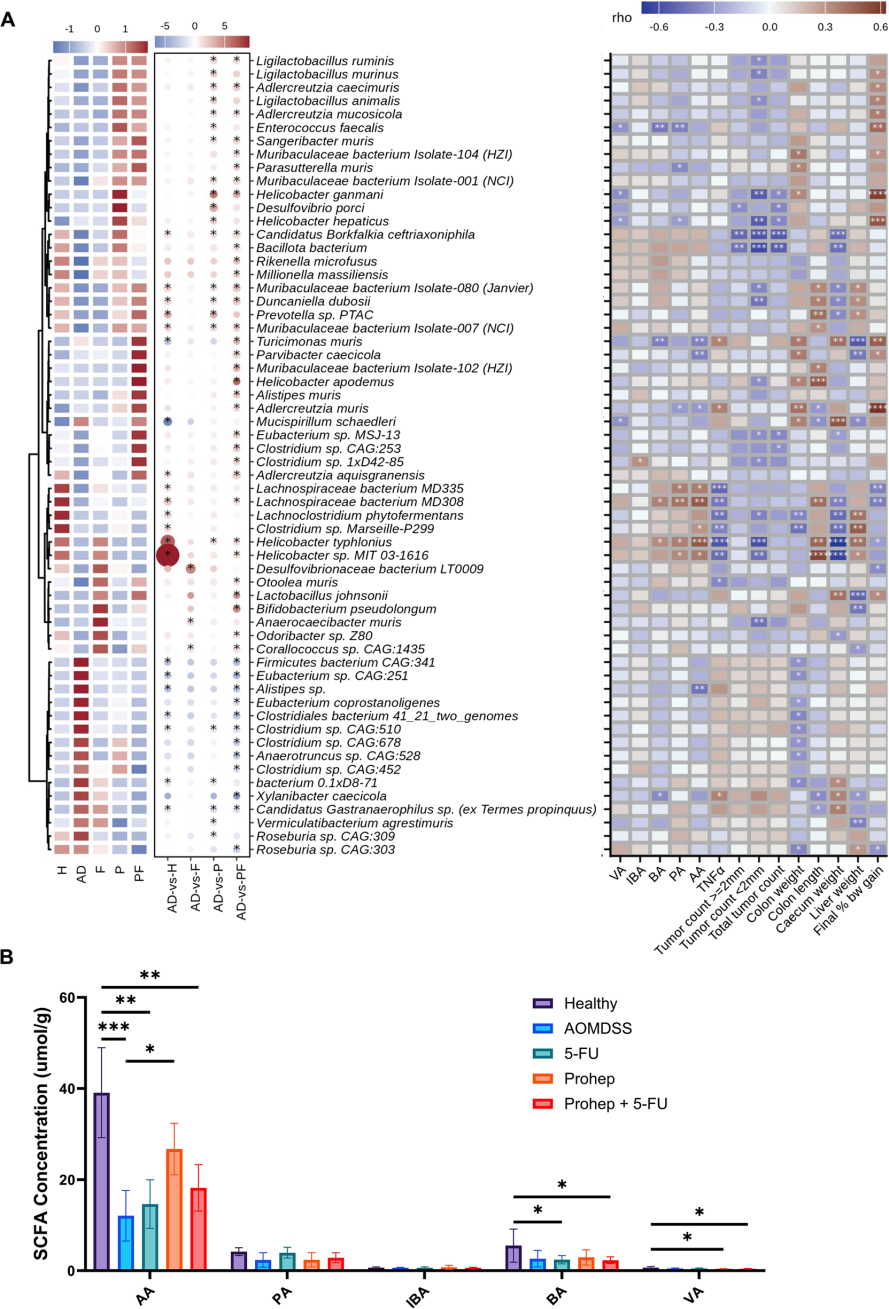
Prohep modulated gut microbiota contributed to alleviate colorectal tumorigenesis and elevated SCFAs concentration. *A* Heatmap showing the *z*-score transformed mean abundance of significantly altered species in H, F, P, or PF as compared to AD (left) and Spearman’s correlation between relative bacterial abundance and fecal SCFA concentrations, colonic cytokine concentration, tumor counts, and colorectal tumorigenesis related organ alterations (right). **B** Fecal concentration of SCFA. AA, acetate; PA, propionate; BA, butyrate; IBA, isobutyrate; and VA, valerate. Differential abundant species based on ANCOM-BC and LDA score (*FDR* < 0.05, fold change > 2 or < 1/2, LDA score (log10) > 2) were labeled with “*” in the right part. *n* = 6–10. * *p* < 0.05; ** *p* < 0.01; *** *p* < 0.001; **** *p* < 0.0001

To assess the impact of Prohep on the gut microbiota of AOM/DSS mice, shotgun metagenomic sequencing was performed. The result indicated a significantly lower alpha diversity (Pielou’s evenness index and Shannon diversity index) in the Prohep + 5-FU group than in the AOM/DSS group (*p* < 0.05) (Supp. Figure 2A). Beta diversity was measured using PERMANOVA analysis, which showed separation of AOM/DSS group from other treatment group, although this was not significant (*R* = 0.1577, *p* = 0.055) (Supp. Figure 2B). To further investigate the specific differences of the gut microbiota composition between groups, the microbial structural profile at the species level was computed (Fig. 5A). The administration of Prohep significantly modulated the abundance of gut microbiota compared to AOM/DSS at the species level. Prohep elevated the abundance of *Ligilactobacillus ruminis*, *Ligilactobacillus murinus*, *Adlercreutzia caecimuris*, *Ligilactobacillus animalis*, *Adlercreutzia mucosicola*, *Enterococcus faecalis*, *Sangeribacter muris*, *Muribaculaceae bacterium Isolate-001 (NCI)*, *Helicobacter ganmani*, *Desulfovibrio porci*, *Helicobacter hepaticus*, *Candidatus Borkfalkia ceftriaxoniphila*, *Muribaculaceae bacterium Isolate-080 (Janvier)*, *Duncaiella dubosii*, *Prevotella* sp. *PTAC*, *Muribaculaceae bacterium Isolate-007 (NCI)*, and *Helicobacter typhlonius* (*p* < 0.05). Besides, Prohep lowered *Clostridum* sp. *CAG:510*, *bacterium 0.1xD8-71*, *Candidatus Gastranaerophilus* sp. *(ex Termes propinquus)*, *Vermiculatibacterium agrestimuris*, and *Roseburia* sp. *CAG:309* (*p* < 0.05) (Fig. 5A).

**Fig. 6:**
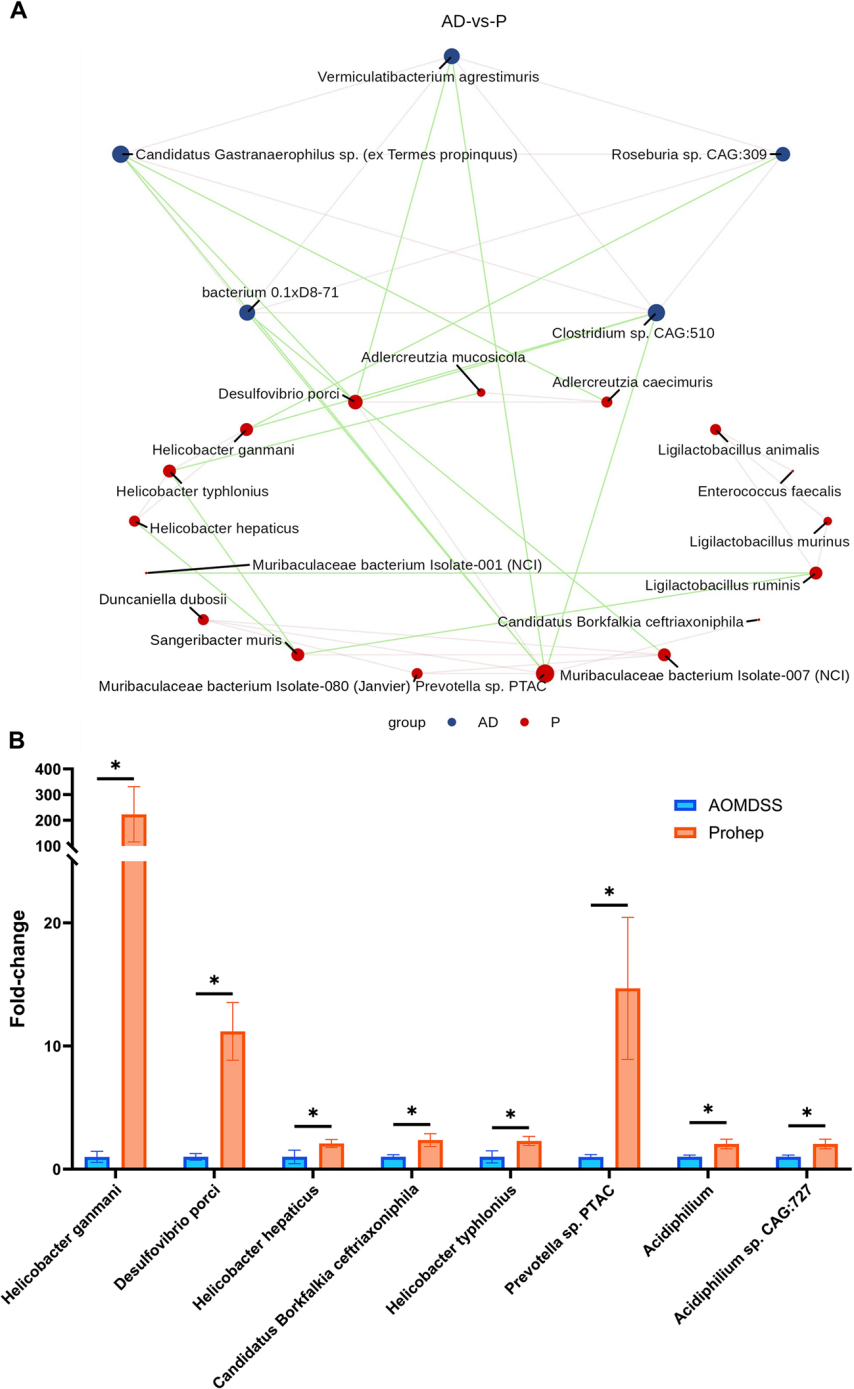
Prohep enriched bacteria negatively correlated with AOM/DSS enriched bacteria. **A** Inner correlation of enriched bacteria in AOM/DSS and Prohep group. Correlations with *p*-value < 0.05 and *r* > 0.3 were considered and visualized in the network. The green edge denotes for negative correlation, and red edge denotes for positive correlation. The size of the node is proportional to the number of connections to other nodes. **B** Fold-change of bacteria enriched by Prohep. *n* = 6–10. * *p* < 0.05; ** *p* < 0.01; *** *p* < 0.001; **** *p* < 0.0001

From the identified bacteria, Spearman’s correlation was carried out to evaluate the inhibitory effects on colorectal tumorigenesis by modulating these bacteria. Prohep elevated *Helicobacter ganmani*, *Desulfovibrio porci*, *Helicobacter hepaticus*, and *Candidatus Borkfalkia ceftriaxoniphila* were found to be inversely correlated to the total tumor count (*p* < 0.05) (Fig. 5A). In particular, *Helicobacter ganmani*, *Helicobacter hepaticus*, and *Candidatus Borkfalkia ceftriaxoniphila* were also inversely correlated to tumor count < 2 mm (*p* < 0.05), and *Desulfovibrio porci* and *Candidatus Borkfalkia ceftriaxoniphila* were inversely correlated to tumor count ≥ 2 mm (*p* < 0.05) (Fig. 5A). Noteworthily, Prohep elevated *Helicobacter typhlonius* inversely correlated to tumor count < 2 mm (*p* < 0.05), and pro-inflammatory cytokines TNF-α (*p* < 0.05), while being positively correlated to the abundance of fecal butyrate, propionate and acetate (*p* < 0.05) (Fig. 5A). A correlation analysis between the enriched species was carried out to study the possible role of bacteria among the microbiota community of AOM/DSS and Prohep. Among the enriched species, *Desulfovibrio porci*, *Prevotella* sp. *PTAC*, *Helicobacter ganmani*, and *Muribaculaceae bacterium Isolate-007 (NCI)* were found to be negatively correlated to AOM/DSS enriched bacteria inducing *Clostridum* sp. *CAG:510*, *bacterium 0.1xD8-71*, *Candidatus gastranaerophilus* sp. (*ex Termes propinquus*), *Vermiculatibacterium agrestimuris* and *Roseburia* sp. *CAG:309* (*p* < 0.05) ***(***Fig. 6A***).*** Among them, *Prevotella sp. PTAC* demonstrated the highest number of correlations with other bacteria and negatively correlated with most of the AOM/DSS enriched bacteria and positively correlated with other Prohep enriched bacteria. In particular, *Prevotella* sp. *PTAC* and *Desulfovibrio porci* were significantly correlated with each other (*p* < 0.05) (Fig. 6A). The enriched *Desulfovibrio porci* was the only species found to be negatively correlated with all AOM/DSS enriched species and total tumor count at the same, highlighting its importance in attenuating CRC gut dysbiosis and tumorigenesis.

**Fig. 7:**
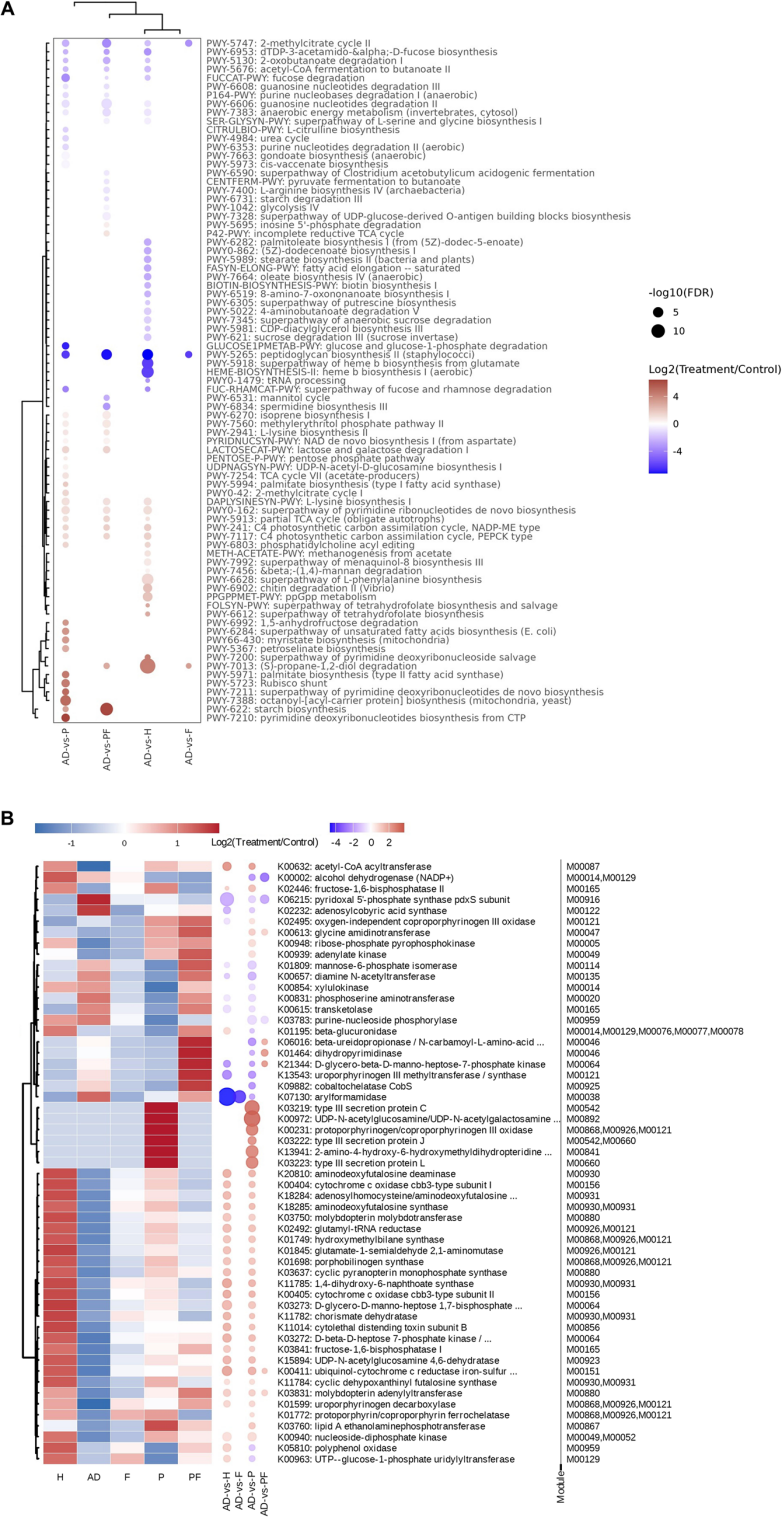
Prohep modulated gut microbiota to induce anti-tumor metabolic functions. **A** Heatmap showing the *z*-score transformed mean abundance of KEGG orthology (KO) originated from three significantly altered KEGG modules in H, F, P, or PF as compared to AD (left). Differential KO based on ANCOM-BC (adjusted *p*-value < 0.1) were displayed in the right part; and **B** significantly altered Metacyc pathway in H, F, T, or TF, as compared to AD. The color is proportional to log2 transformed fold change (treatment/control), while the size is proportional to the value of -log10 transformed adjusted *p* (*FDR*-corrected). *n* = 6–10. * *p* < 0.05; ** *p* < 0.01; *** *p* < 0.001; **** *p* < 0.0001

Furthermore, the possible functions of Prohep on governing metabolic and biosynthesis pathways in comparison with AOM/DSS were studied from Metacyc and KEGG analysis. For the Metacyc analysis, Prohep downregulated 17 pathways and upregulated 26 pathways (Fig. 7A). In particular, Prohep reduced peptidoglycan biosynthesis II (staphylococci) (*p* < 0.05) and dTDP-3-acetamido-α-D-fucos biosynthesis (*p* < 0.05), which were related to the biosynthesis of the detrimental peptidoglycan and lipopolysaccharide (LPS). Prohep also downregulated purine nucleotides degradation II (aerobic) (*p* < 0.05), which limits the conversion of purine into cancer related uric acid. In addition, Prohep elevated pathways related to the biosynthesis of beneficial compounds, including L-lysine biosynthesis I (*p* < 0.05) and L-lysine biosynthesis II (*p* < 0.05) which were related to the biosynthesis of L-lysine; octanoyl-[acyl-carrier protein] biosynthesis (mitochondria and yeast) (*p* < 0.05) for the biosynthesis of lipoic acid; pyrimidine deoxyribonucleotides biosynthesis from CTP (*p* < 0.05), superpathway of pyrimidine deoxyribonucleotides de novo biosynthesis (*p* < 0.05), and superpathway of pyrimidine ribonucleotides de novo biosynthesis (p < 0.05) for the biosynthesis of pyrimidine; Rubisco shunt (*p* < 0.05) and 2-methylcitrate cycle I (*p* < 0.05) for SCFA precursor pyruvate; and palmitate biosynthesis II (bacteria and plants) (*p* < 0.05), superpathway of unsaturated fatty acids biosynthesis (*Escherichia coli*) (*p* < 0.05) and palmitate biosynthesis I (animals and fungi) (*p* < 0.05) which related to palmitate biosynthesis. Furthermore, Prohep also upregulated pathways energy utilization pathway of lactic-acid producing bacteria, lactose and galactose degradation I (*p* < 0.05) and TCA cycle VII (acetate-producers) (*p* < 0.05) which indicates for greater abundance of acetic acid producing bacteria.

In terms of the KEGG analysis, Prohep downregulated 23 KO gene and upregulated 55KO gene (Fig. 7B). Especially, Prohep decreased arylformamidase (*p* < 0.05), phosphoserine aminotransferase (*p* < 0.05), beta-glucuronidase (*p* < 0.05), alcohol dehydrogenase (NADP +) (*p* < 0.05), transketolase (*p* < 0.05) and purine-nucleoside phosphorylase (*p* < 0.05). In addition, Prohep increased aminodeoxyfutalosine deaminase (*p* < 0.05), aminodeoxyfutalosine synthase (*p* < 0.05), 1,4-dihydroxy-6-naphthoate synthase (*p* < 0.05), chorismate dehydratase (*p* < 0.05), cyclic dehypoxanthinyl futalosine synthase (*p* < 0.05), adenosylhomocysteine/aminodeoxyfutalosine nucleosidase (*p* < 0.05), acetyl-CoA acyltransferase (*p* < 0.05), hydroxymethylbilane synthase (*p* < 0.05), porphobilinogen synthase (*p* < 0.05), glutamate-1-semialdehyde 2,1-aminomutase (*p* < 0.05), glutamyl-tRNA reductase (*p* < 0.05), fructose-1,6-bisphosphatase I (*p* < 0.05), fructose-1,6-bisphosphatase II (*p* < 0.05), and 2-amino-4-hfigureydroxy-6-hydroxymethyldihydropteridine diphosphokinase/dihydropteroate synthase (*p* < 0.05).

The fecal SCFA concentration was accessed based on the results of Metacyc analysis. In comparison with the healthy group, the fecal acetate concentration was lowered in the AOM/DSS, 5-FU and Prohep + 5-FU groups (*p* < 0.05) (Fig. 5B). Meanwhile, the fecal acetate concentration in Prohep was significantly increased compared to the AOM/DSS group (*p* < 0.05). Furthermore, the relative abundance of acetate-producing *Acidiphilium* genus and *Acidiphilium* sp. *CAG:727* were upregulated by Prohep in comparison to AOM/DSS (*p* < 0.05).

## Discussion

In this study, Prohep significantly alleviated AOM/DSS-induced colorectal tumorigenesis by suppressing proliferative STAT3, inducing apoptotic p53, modulating the gut microbiota and elevating acetate concentration. Prohep also demonstrated the strongest alleviative effects compared to 5-FU, and Prohep + 5-FU. In the current study, all experimental mice developed colorectal tumors from the AOM/DSS treatment. Meanwhile, some mice in AOM/DSS, 5-FU, and Prohep + 5-FU groups reached humane endpoints that required euthanasia as determined by animal facility veterinarians. Notably, only Prohep significantly improved the survivability of experimental animals, but not in 5-FU and Prohep + 5-FU groups. Although all three treatment groups, including 5-FU, Prohep, and Prohep + 5-FU, significantly reduced the total tumor count; only Prohep significantly reduced the total tumor size as well. 5-FU was the only group that was not able to reduce the enlarged caecum. In addition, 5-FU and Prohep + 5-FU led to a reduction in liver weight in comparison to the healthy group. As the liver is the major organ for metabolizing 5-FU, the administration of 5-FU was suggested to cause liver function failure and hepatotoxicity including apoptosis, inflammation, and collagen fibrosis, as seen with the reduced liver weight [39–41]. Prohep was the only group that significantly improved survivability and reduced tumor count, size, and caecum weight when compared to the AOM/DSS group. Therefore, it was suggested that Prohep was an effective therapeutic alternative, as it protected liver functions while also being anti-CRC.

In this study, Prohep demonstrated anti-inflammatory responses in the colon. Inflammation-related histopathological parameters, including hyperplasia, disrupted colonic structure, lengthened colonic crypt depth, and collagen fibrosis, were all alleviated by Prohep. In addition, the pro-inflammatory cytokines TNF-α and inflammatory regulator STAT3 activation were suppressed. Besides the well-known pro-inflammatory IL-6 cytokine, TNF-α was found to promote proliferation and inhibit apoptosis in colon cancer cells by activating STAT3 [42]. STAT3 was suggested to be a mediator connecting inflammation and cancer progression, in which silencing of the STAT3 was found to limit CRC cell growth and induce cell death at the G2/M stage [43, 44]. Prohep also downregulated c-jun and c-fos expression levels which functioned as the effectors of STAT3-induced tumor cell proliferation [45]. Furthermore, STAT3 silencing also led to apoptosis through upregulation of p53 and caspase-3 [44]. Similar results were observed in the present study with the elevated expression level of p53 and c-casp3. p53 serves a critical function in suppressing tumor growth by inducing cell cycle arrest, DNA repair, senescence and apoptosis [46]. Deficiencies of p53 were often found in cancer, and the restoration of p53 was targeted for cancer therapy including CRC [46, 47]. At the same time, p53 also inhibits STAT3 activators and attenuates STAT3 functions [48]. Given both STAT3 and p53 are effective targets and the presence of opposing STAT3 − p53 regulatory loops, co-targeting STAT3 and p53 by Prohep was therefore suggested to be promising in CRC regulation.

Apart from its effects on cell signaling pathways, Prohep administration also altered the gut microbiota. Prohep enriched the relative abundance of *Helicobacter ganmani*, *Desulfovibrio porci*, *Helicobacter hepaticus*, *Candidatus Borkfalkia ceftriaxoniphila*, and *Helicobacter typhlonius*, which showed a reverse correlation with colorectal tumor counts in this study. *Helicobacter ganmani* and *Helicobacter hepaticus* are two *Helicobacter* species naturally colonizing mouse gut which are reported to be related to IBD development [49, 50]. Nevertheless, a recent study suggested that *Helicobacter ganmani* and the augment of *Helicobacter* enhanced the induction of RORγt^+^ Foxp3^+^ iTregs cells which improved the resistance against DSS-induced colitis in a fecal microbiota transplant (FMT) model pretreated with glycerol monolaurate [51]. Further studies on the potential beneficial effects of *Helicobacter ganmani* in the colon are necessary to reconcile the contradicting observations. Similarly, a previous study described that *Helicobacter typhlonius* when co-administrated with *Akkermansia muciniphila* would reduce intestinal tumors in *Apc* mutant mice, while opposing results were observed when either bacterium was singly administrated [52]. Moreover, *Desulfovibrio porci* was also found to be inversely correlated to tumor count. Except for identifying it as a hydrogen sulfide (H_2_S) producing species, its effects on animals were not well-documented [53–56]. H_2_S was reported with dual effects on cancer cells following a biphasic dose–response curve [57, 58]. Recent studies suggest that H_2_S exposure in high concentrations for prolonged periods induced apoptotic responses in cancer cells both in vivo and in vitro [57, 59]. Thus, it was proposed that the H_2_S-producing properties of *Desulfovibrio porci* might contribute to the alleviation of colorectal tumorigenesis in this study which is worth further validation in the future. Furthermore, the enriched *Candidatus Borkfalkia ceftriaxoniphila,* a newly discovered and low abundant commensal species, had a significant increase in abundancies accompanying the growth of probiotics in the human gut [60].

The inner correlation analysis identified Prohep-enriched *Desulfovibrio porci*, *Prevotella* sp. *PTAC*, *Helicobacter ganmani*, and *Muribaculaceae bacterium Isolate-007 (NCI)* were inversely correlated to AOM/DSS enriched bacteria. Both *Desulfovibrio porci* and *Prevotella* sp. *PTAC* displayed a significant role in Prohep-induced gut microbiota modulation and a positive correlation in relative abundance was found between them. A higher abundance of *Prevotella* was reported to be linked to lower risks of CRC progression in CRC patients [61]. Although *Prevotella* sp. *PTAC* was not found to be significantly correlated to reduced tumor count in this study, its co-occurrence with *Desulfovibrio porci* and other Prohep-enriched bacteria suggested it played a vast role in supporting the growth of these beneficial bacteria in CRC management. Notably, only the enrichment of *Desulfovibrio porci* negatively correlated to all AOM/DSS enriched bacteria. Given its negative correlation with CRC tumor count as well, the upregulation of *Desulfovibrio porci* was suggested to be crucial in alleviating CRC tumorigenesis through reversing AOM/DSS-induced gut microbiota.

Apart from the correlation against the colorectal tumorigenesis-related parameters, the mechanisms behind the anti-CRC effects of Prohep was also studied through the prediction of the metabolic functions from Metacyc and KEGG analysis. From the Metacyc analysis, Prohep downregulated the CRC-related peptidoglycan and LPS biosynthesis as well as the conversion of uric acid from purine. Peptidoglycan is a major component of the gram-positive bacteria cell wall [62, 63]. When peptidoglycan is transferred across the intestinal epithelial cells, it interacts with macrophages, triggers IL-6 secretion and induces inflammation and fibrosis in Crohn’s disease and CRC [64, 65]. Similarly, LPS is a cellular component from bacterial membranes which is known for its endotoxic properties [66, 67]. LPS would induce inflammation by activating nuclear factor-κB (NF-κB) pathway via Toll-like receptor 4 (TLR4) which exacerbates gut barrier dysfunction and promotes CRC development [68–70]. In addition, in the catabolism of purine nucleotides, uric acid is released which exhibits a positive association with CRC incidence [71, 72].

Prohep, on the other hand, elevated the biosynthesis pathways of beneficial compounds including L-lysine, lipoic acid, pyrimidine, and palmitate. While L-lysine does not contribute directly to cancer management, it could be completely be converted into butyrate and acetate by gut microbiota like *Intestinimonas* strain AF211 and served a significant role in inhibiting CRC and maintaining the growth of normal colonocytes [73, 74]. Meanwhile, the combination of lysine with epigallocatechin gallate (EGCG) were found to enhance the inhibitory effects of EGCG on the growth of colon cancer cell line HCT 116 [75]. Alpha-lipoic acid (LA) was found to have anti-CRC by triggering cell death in tumor cells and could be combined with doxorubicin or 5-FU to synergistically kill CRC cells [76, 77]. LA also prevent inflammatory and oxidative responses by suppressing the NF-κB pathway, as well as downregulating TNF-α, IL-6, COX-2, MDA, and MPO [78]. Pyrimidine and its derivatives were reported with pharmacological and anti-cancer properties, in which pyrimidine metabolic pathways were reported to regulate the chemotherapeutic effects of chemo drugs like 5-FU, tegafur, and thioguanine [79–81]. In particular, the derivative N-[2-(dimethylamino)ethyl]−2,3-dimethyl-4-oxo-4H-pyrido[1,2-a]thieno[2,3-d]pyrimidine-9-carboxamide (PTP) showed strong anti-tumor effects against human CRC cells by activating p53. Palmitate is one of the common saturated fatty acids found in animals and plants which previous studies reported that a low intake of palmitic acid showed inverse associations with CRC, and palmitic acid alongside ceramide were strong inhibitors of the EMT signaling axis of colorectal cancer cells [82, 83]. The upregulation of Rubisco shunt and 2-methylcitrate cycle II both supported the production of pyruvate which is a precursor of acetate and butyrate [84, 85]. Through pyruvate decarboxylation, pyruvate is oxidated to the intermediate acetyl-CoA, which could produce acetate via acetate kinase or condense with another acetyl-CoA and be further reduced for butyrate [86–88].

Moreover, Prohep elevated metabolic pathways related to energy utilization of lactic acid-producing bacteria (LAB) and acetate producers. Since Prohep is composed mostly of LAB, the increase of lactose and galactose degradation was expected, and intestinal galactose was reported to have a protective effect against CRC by inhibiting mucosal proliferation [89]. Besides, the TCA cycle of acetic acid bacteria was enhanced by Prohep. Acetic acid bacteria are known for their properties in oxidizing ethanol to acetic acid and the abundance of acetate-producing *Acidiphilium* was elevated [90]. Acetate was identified as one of the important metabolites which inhibited CRC tumorigenesis in both in vivo and in vitro models [91–93]. Acetate reduced the tumor size of a CRC-cell-injected xenograft mouse model [91]. It was suggested that acetate enhanced growth arrest and apoptosis of CRC cells through increasing oxygen consumption and reactive oxygen species production [92, 93]. Previous studies demonstrated more pronounced apoptotic effects on CRC cells compared to normal colonocytes, achieved by enhancing MCT1, MCT4, and CD147 while also re-localizing MCT1 at the plasma membrane [94, 95]. Also, acetate would serve as an energy substrate for normal colonocytes via de novo lipogenesis and allows the normal cells to outcompete cancer cells, which primarily rely on glycolysis for energy production [96]. Given the elevation of pathways related to SCFAs and acetate production from Metacyc analysis, the fecal SCFA profile was evaluated. Prohep significantly enriched acetate concentration in this study. Similar to our previous study, Prohep increased fecal acetate level to achieve hepatic lipid regulation in the MASLD/MASH model [22]. Our results suggest that acetate might play a role in the overall reduction of colorectal tumorigenesis.

In terms of the KEGG analysis, Prohep downregulated the detrimental arylformamidase, phosphoserine aminotransferase, beta-glucuronidase, alcohol dehydrogenase (NADP +), transketolase, and purine-nucleoside phosphorylase pathways. Arylformamidase (AFMID) was involved in the conversion of tryptophan into kynurenine by the transcription factor MYC in both cultured CRC cells and CRC patients [97, 98]. Phosphoserine aminotransferase is responsible for serine biosynthesis and an elevated serine was suggested to enhance the growth of CRC cells [99]. The overexpression of phosphoserine aminotransferase was reported to heighten CRC cells chemoresistance and cell growth [100]. The serum activity of beta-glucuronidase was found to be higher in CRC patients than in healthy subjects, which suggested it as a marker of CRC [101]. In the ascorbate-dependent alcohol oxidation system, alcohol dehydrogenase (NADP +) facilitated oxygen to be consumed and hydrogen peroxide (H_2_O_2_) to form [102]. The level of H_2_O_2_ in the tumor microenvironment was closely associated with the development of CRC [103]. Transketolase was abnormally increased in CRC which promoted cancer cell glycolysis by enhancing AKT phosphorylation and eventually worsening CRC metastasis [104]. Purine-nucleoside phosphorylase (PNP) was identified as a cancer marker as plasma PNP levels on average four times higher in cancer patients [105]. For CRC, the expression level of PNP was also correlated with lymph vessel invasion, positive lymph node metastasis, and advanced stage of CRC [106]. The downregulation of these CRC-related pathways strengthened Prohep’s promising effects in inhibiting colorectal tumorigenesis.

Moreover, Prohep elevated multiple beneficial pathways in the regulation of CRC. Aminodeoxyfutalosine deaminase, aminodeoxyfutalosine synthase, 1,4-dihydroxy-6-naphthoate synthase, chorismate dehydratase, cyclic dehypoxanthinyl futalosine synthase, and adenosylhomocysteine/aminodeoxyfutalosine nucleosidase are related to menaquinones which are the bacterial forms vitamin K produced by gut microbiota [107]. Menaquinone or vitamin K2 was found to reduce KRAS proliferation in colon cancer cells and promote apoptotic cell death in CRC mice [108, 109]. Acetyl-CoA acyltransferase (ACAA) was shown to have a negative correlation with the resistance of the targeted cancer drug, cetuximab, in CRC which the overexpression of ACAA would suppress proliferation and lower cetuximab tolerance in CRC cells [110]. Hydroxymethylbilane synthase was identified as a tumor suppressor gene, and its inactivation was identified in patients with intermittent porphyria and sporadic HCC [111, 112]. Porphobilinogen synthase is the crucial first step of tetrapyrrole biosynthesis which tetrapyrroles like unconjugated bilirubin, bilirubin ditaurate, biliverdin, biliverdin-/bilirubin dimethyl ester, urobilin, stercobilin, and protoporphyrin exhibited DNA-damaging and apoptosis in colon and liver cancer cells [113, 114]. Meanwhile, porphobilinogen synthase, along with glutamate-1-semialdehyde 2,1-aminomutase and glutamyl-tRNA reductase are involved in the production and utilization of aminolevulinic acid (ALA) which was shown to inhibit CRC cells [115–117]. Fructose-1,6-bisphosphatase I could inactive NF-κB which suppresses CRC and similarly, fructose-1,6-bisphosphatase II was found to regulate gastric cancer in an inversed relationship [118, 119]. 2-Amino-4-hydroxy-6-hydroxymethyldihydropteridine diphosphokinase/dihydropteroate synthase was required in the biosynthesis of tetrahydrofolate which was associated with lowered risk of serrated polyps in CRC [120]. These results suggested that the gut microbiota modulation of Prohep was important in establishing an anti-CRC metabolic profile.

In current study, Prohep was found to be the most effective treatment for AOM/DSS mice when compared with 5-FU and Prohep + 5-FU. No additive or synergistic effects were observed when Prohep was given in adjuvant with 5-FU. Prohep presented the strongest inhibition on colorectal tumorigenesis and inflammation among all groups. In the present study, even though 5-FU reduced the total tumor count in the colon, it failed to reduce the level of p-STAT3 and upregulate that of p53. The activation of STAT3 was reported to promote 5-FU resistance in CRC through increasing Mcl-1-dependent cytoprotective autophagy, by which 5-FU resistant cells would transfer p-STAT3-containing exosomes to the recipient cells and induce chemoresistance against 5-FU [121, 122]. On the other hand, the loss or deficiency of p53 would contribute to 5-FU resistance and detriment the DNA damaging effects of 5-FU against CRC cells [123, 124]. The restoration of p53 was reported to significantly improve 5-FU sensitivity in CRC cells [125]. Nonetheless, even though Prohep increased p53 and reduced p-STAT3, the effects were not displayed when Prohep was co-treated with 5-FU. Besides a disrupted gut microbiome environment, 5-FU could lead to gut dysbiosis and intestinal mucositis. These effects may hinder the adhesion, colonisation and activation of probiotic species and might thus limit the anti-CRC effects of Prohep when co-administrated [21, 126, 127].

## Conclusion

In conclusion, our findings demonstrated that Prohep reduced AOM/DSS-induced CRC carcinogenesis. Through suppressing STAT3, and activating apoptotic-inducing p53, Prohep significantly reduced total tumor count, total tumor size, cecum weight, colonic crypt depth, colonic inflammation, and collagen fibrosis induced by AOM/DSS. Furthermore, Prohep enriched the abundance of beneficial bacteria and acetate level which contributed to combat CRC. Notably, Prohep showed superior anti-tumorigenesis effects compared to both 5-FU alone and Prohep + 5-FU in the treatment of CRC. These findings indicate the potential for Prohep to be an effective alternative treatment for CRC.

## Supplementary Information

Below is the link to the electronic supplementary material.

Supplementary file1 (DOCX 2202 KB)

## Data Availability

Data will be made available on request.
