# Supplementary material for: Probiotic Mixture Attenuates Colorectal Tumorigenesis in Murine AOM/DSS Model by Suppressing STAT3, Inducing Apoptotic p53 and Modulating Gut Microbiota
Source: Probiotics Antimicrob Proteins. 2024 Dec 6;17(4):2227–43. doi: 10.1007/s12602-024-10405-1 (PMC12405048; doi:10.1007/s12602-024-10405-1)
Supplement: Supplementary file 1 — Supplementary file1 (DOCX 2202 KB) [file 12602_2024_10405_MOESM1_ESM.docx]

**Probiotic mixture attenuates colorectal tumorigenesis in murine AOM/DSS model by suppressing STAT3, inducing apoptotic p53 and modulating gut microbiota**

**Hoi Kit Matthew Leung ^a 1^, Emily Kwun Kwan Lo ^a 1^, Congjia Chen ^a^, Fangfei Zhang ^a^, Felicianna ^a^, Marsena Jasiel Ismaiah ^a^, Hani El-Nezami ^a b *^**

1. School of Biological Sciences, University of Hong Kong, Pokfulam 999077, Hong Kong, China
2. Institute of Public Health and Clinical Nutrition, School of Medicine, University of Eastern Finland, FI-70211 Kuopio, Finland

- Correspondence: elnezami@hku.hk, Hani El-Nezami

1. Equally contributed to the manuscript.

***Supplementary Figure 1. Full blot images of western blot data.***


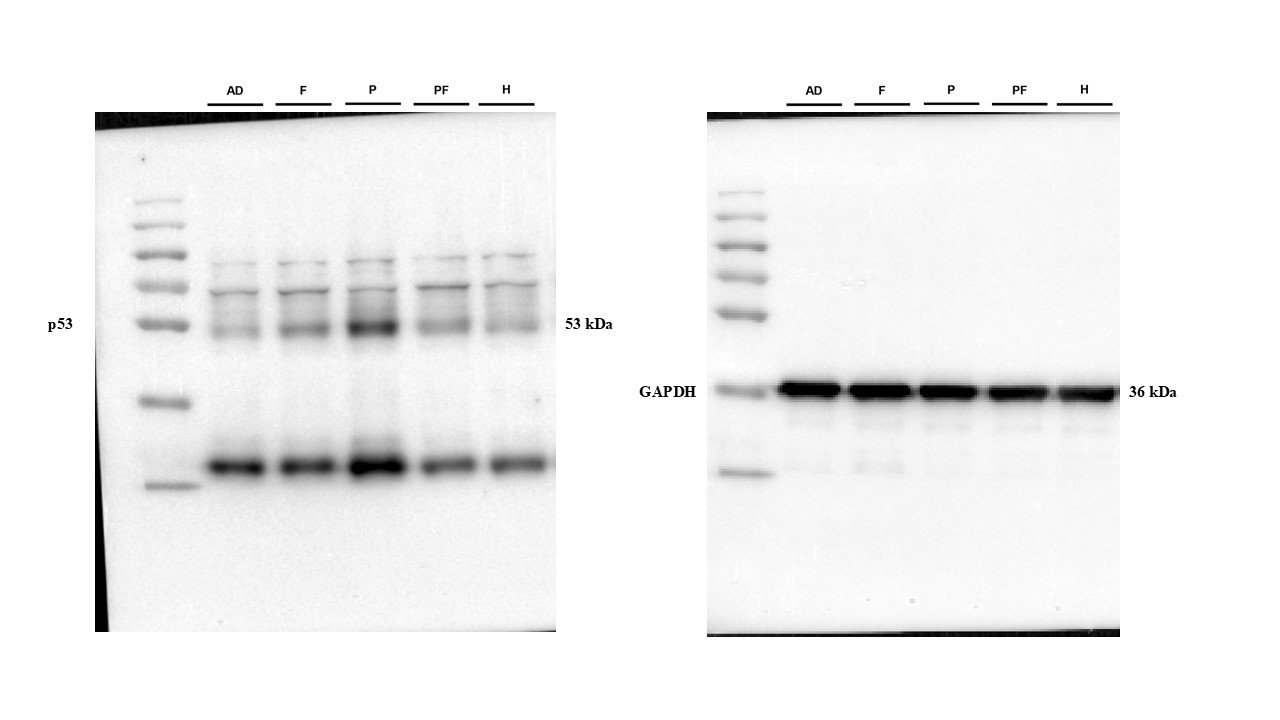


***Supplementary Figure 2. (A) Alpha diversity analysis of bacteria between groups. (B) Beta diversity analysis of bacterial between groups. n = 6-10. *p < 0.05; **p < 0.01; ***p < 0.001.***
